# p38 MAPK as a gatekeeper of reprogramming in mouse migratory primordial germ cells

**DOI:** 10.3389/fcell.2024.1410177

**Published:** 2024-06-06

**Authors:** Daiji Okamura, Aoi Kohara, Yuta Chigi, Tomoka Katayama, Jafar Sharif, Jun Wu, Yumi Ito-Matsuoka, Yasuhisa Matsui

**Affiliations:** ^1^ Department of Advanced Bioscience, Faculty of Agriculture, Kindai University, Nara, Japan; ^2^ Institute of Advanced Medical Sciences, Tokushima University, Tokushima, Japan; ^3^ Laboratory for Developmental Genetics, RIKEN Center for Integrative Medical Sciences (IMS), Yokohama, Japan; ^4^ Department of Molecular Biology, University of Texas Southwestern Medical Center, Dallas, TX, United States; ^5^ Hamon Center for Regenerative Science and Medicine, University of Texas Southwestern Medical Center, Dallas, TX, United States; ^6^ Cell Resource Center for Biomedical Research, Institute of Development, Aging and Cancer, Tohoku University, Sendai, Japan; ^7^ Graduate School of Life Sciences, Tohoku University, Sendai, Japan; ^8^ Graduate School of Medicine, Tohoku University, Sendai, Japan

**Keywords:** p38 MAPK, migratory primordial germ cells, teratoma, reprogramming, EGCs, pluripotency, gatekeeper

## Abstract

Mammalian germ cells are derived from primordial germ cells (PGCs) and ensure species continuity through generations. Unlike irreversible committed mature germ cells, migratory PGCs exhibit a latent pluripotency characterized by the ability to derive embryonic germ cells (EGCs) and form teratoma. Here, we show that inhibition of p38 mitogen-activated protein kinase (MAPK) by chemical compounds in mouse migratory PGCs enables derivation of chemically induced Embryonic Germ-like Cells (cEGLCs) that do not require conventional growth factors like LIF and FGF2/Activin-A, and possess unique naïve pluripotent-like characteristics with epiblast features and chimera formation potential. Furthermore, cEGLCs are regulated by a unique PI3K-Akt signaling pathway, distinct from conventional naïve pluripotent stem cells described previously. Consistent with this notion, we show by performing *ex vivo* analysis that inhibition of p38 MAPK in organ culture supports the survival and proliferation of PGCs and also potentially reprograms PGCs to acquire indefinite proliferative capabilities, marking these cells as putative teratoma-producing cells. These findings highlight the utility of our *ex vivo* model in mimicking *in vivo* teratoma formation, thereby providing valuable insights into the cellular mechanisms underlying tumorigenesis. Taken together, our research underscores a key role of p38 MAPK in germ cell development, maintaining proper cell fate by preventing unscheduled pluripotency and teratoma formation with a balance between proliferation and differentiation.

## Introduction

Germ cells provide a stable and continuous link between generations essential for the continuity of species. In mammals, germline development is a highly dynamic and exquisitely choreographed process that involves numerous transitionary steps. In mice, primordial germ cells (PGCs), the common precursor of oocytes and spermatozoa, are specified from competent epiblast cells receiving inductive signals from the extra-embryonic ectoderm (Lawson et al., 1999; Ohinata et al., 2009). Thereafter, PGCs increase in number while migrating through the developing hindgut and finally colonize the emerging gonads, where they differentiate into either oocytes or spermatozoa. Among vertebrates such as fish (Gross-Thebing et al., 2017), frogs (Wylie et al., 1985), salamanders (Chatfield et al., 2014), and even mice (Mikedis and Downs, 2012; Mikedis and Downs, 2017), migratory PGCs are not yet irreversibly committed, but instead retain a broad developmental potential; whereas the fate of germ cells are determined after PGCs colonize the gonads (Nicholls et al., 2019). As a result, migratory PGCs can form teratomas when transplanted to ectopic sites (Stevens, 1964) and be reprogrammed to pluripotent cell types without introduction of exogenous reprogramming genes (Matsui et al., 1992; Resnick et al., 1992; Shamblott et al., 1998) required for derivation of induced pluripotent stem cells (iPSCs) (Takahashi and Yamanaka, 2006; Takahashi et al., 2007). This could in part be mediated by a “latent” rather than a “silenced” pluripotent network (Leitch and Smith, 2013; Nicholls and Page, 2021; De Felici et al., 2021). Specifically, a sub-group of PGCs could be converted into pluripotent embryonic germ cells (EGCs) in primary culture via combinatory treatments with leukemia inhibitory factor (LIF), fibroblast growth factor-2 (FGF2), and stem cell factor (SCF) (Matsui et al., 1992; Resnick et al., 1992), while the majority of the remaining PGCs undergo apoptotic cell death (Pesce and De Felici, 1994). Such phenotypes of primary cultured PGCs may mimic the fate of PGCs that deviate from their route toward emerging gonads and instead form germ cell tumors (teratoma) *in vivo* (Runyan et al., 2008; Müller et al., 2021). In addition, treatment with 2i (PD0325901 and CHIR99021), which increases the efficiency of iPSC production (Shi et al., 2008; Silva et al., 2008), can replace FGF2 and SCF during derivation of EGCs from migrating PGCs at E8.5 (Leitch et al., 2010), indicating that cytokine signaling or epigenetic modifications driven by combination of these growth factors and chemical compounds may reprogram PGCs to a pluripotent state (Sekita et al., 2016).

It is, however, unknown whether direct manipulation of certain cytokine signalings and epigenetic modifiers by chemical compounds could endow pluripotent features to PGCs without exogenous growth factors such as LIF. Such a simplistic approach should be useful to understand the mechanisms governing pluripotency or teratoma formation in PGCs. Of note, the PI3K-Akt pathway activated by LIF is essential for the maintenance of naïve pluripotency (Watanabe et al., 2006; Niwa et al., 2009; Takahashi et al., 2005), and signaling of which is also involved in germ cell reprogramming (Kimura et al., 2003; Matsui et al., 2014). These imply that the PI3K-Akt pathway might remain dominant unless LIF supplementation is withdrawn, potentially preventing the activation of other cascades involved in reprogramming. Therefore, achieving of LIF-independent reprogramming of PGCs by chemical compounds may be an approach for novel molecules involving reprogramming to pluripotency that PGCs potentially contain.

Mitogen-activated protein kinase (MAPK) are serine/threonine protein kinases that are classified mainly into three groups: extracellular signal-regulated kinase 1/2 (ERK1/2), c-Jun amino (N)-terminal kinase (JNK), and p38 MAPK. p38 MAPK plays important roles in various cellular processes, including inflammation, stress response, cell differentiation, apoptosis (cell death), and human diseases (Zarubin and Han, 2005; Cuenda and Rousseau, 2007). In particular, p38 MAPK plays a critical role in cell transformation by inhibiting cell cycle progression. In addition, p38 MAPK possesses pro-apoptotic effects, which likely helps prevention of cancerous cellular transformation (Phan et al., 2023). In the context of germ cell development, p38 MAPK plays a pivotal role in controlling progression of germ cells differentiation, particularly in males (Luo et al., 2022). However, the role of p38 MAPK in migratory PGCs remains to be elucidated.

In this study, to identify chemical compounds that may reprogram PGCs, we investigated the effect of p38 mitogen-activated protein kinase (p38 MAPK) inhibitors on freshly isolated hindgut encapsulating migrating PGCs at E8.75. Strikingly, while no self-renewing cells could be obtained in control medium, treatment with p38 MAPK inhibitor alone could give rise to stable pluripotent cell lines, termed chemically induced EG-like cells (cEGLCs) that could be expanded indefinitely *in vitro* and exhibited unique naïve pluripotency characterized by chimera formation ability and independency to exogenous factors such as LIF and FGF2/Activin-A. cEGLCs, in part, represent a naïve pluripotent state with epiblast-like features that differ from conventional EGCs. In addition, we established a model of teratoma formation using mouse migratory PGCs *in vitro* and *ex vivo* via treatment with chemical compounds, and taking advantage of this model identified p38 MAPK as a gatekeeper of reprogramming in mouse migratory primordial germ cells.

## Materials and methods

### Mice

To obtain embryos, ICR females (Charles River Laboratories) were mated with Oct4-deltaPE-GFP transgenic male mice on the B6D2F1 background (Yoshimizu et al., 1999). Female mice were used between 6 and 25 weeks of age. All the animal experiments were performed under the ethical guidelines of the Kindai University, and animal protocols were reviewed and approved by the Animal Care and Use Committee of Kindai University and the Tohoku University Animal Studies Committee.

### Effect of p38 MAPK inhibitor(s) on the survival of cultured PGCs

**FIGURE 1:**
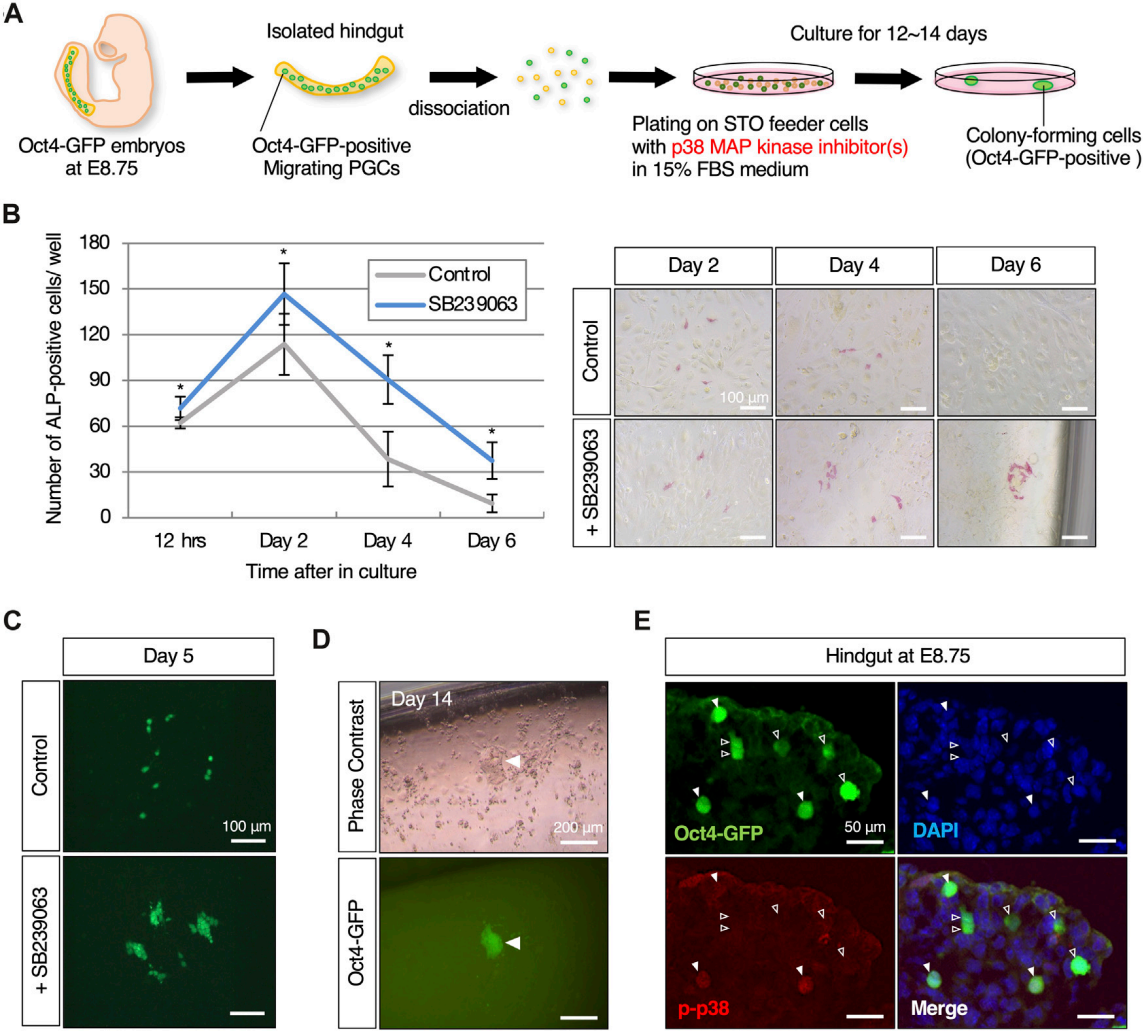
p38 MAPK, which is exclusively expressed in its phosphorylated form in migratory mouse PGCs, induces reprogrammed colony-forming cells through its inhibition **(A)** Schematic representation of the experimental workflow in induction of reprogramming in mouse migratory PGCs with p38 MAPK inhibitor. Isolated hindgut encapsulating Oct4-GFP-expressing migratory PGCs at E8.75 is dissociated, and then plated on STO-feeder cells with p38 MAPK inhibitor-contained medium. Colony-forming cells which are positive for Oct4-GFP expression are emerged after 12–14 days in culture. **(B)** Graphical representation of the change in the number of ALP (alkaline phosphatase)-positive cultured PGCs with or without the p38 MAPK inhibitor, SB239063, and the images at the indicated culture date. Error bars indicate s.d. (*n* = 3, biological replicates). The two-way ANOVA results show that there is a significant effect of the group (*p* = 0.0031). Scale bar, 100 μm. **(C)** Fluorescence images of dispersed (control) and clustered (with SB239063) Oct4-GFP-positive cultured PGCs on day 5. Scale bar, 100 μm. **(D)** Images of Oct4-GFP-positive colony-forming cells that appeared approximately 12–14 days after plating. White arrowheads indicate Oct4-GFP-positive cells that formed a colony at the edge of the well. Scale bar, 200 μm. **(E)** Immunofluorescence of phosphorylated p38 MAPK in cryosection of hindgut isolated from Oct4-GFP embryos at E8.75. White arrowheads indicate Oct4-GFP-positive migrating PGCs that exclusively express the phosphorylated p38 MAPK in the hindgut (filled arrowheads), and not express (empty arroheads). Scale bar, 50 μm.

Oct4-deltaPE-GFP transgenic male mice were mated with ICR female mice in the afternoon and checked for the presence of vaginal plugs the following day. The day on which a plug was found was E0.5. The developing hindgut encapsulating migrating PGCs in an embryo at E8.75 was surgically isolated with a tungsten needle in cold Dulbecco’s Modified Eagle’s Medium (DMEM_high glucose, L-glutamine, and sodium pyruvate plus) (nacalai tesque, 08458-16) supplemented with 10% Fetal Bovine Serum (FBS) (Gibco, 10270-106) and Penicillin-Streptomycin solution (P/S) (1x) (Wako, 168-23191). An isolated hindgut was dissociated with 0.25% trypsin/1 mM EDTA for 10 min at 37°C and then plated on mitomycin C (MMC) (FUJIFILM, 139-18711)-inactivated STO feeders in single well of a 4-well plate (SPL Life Sciences, SPL-30004) in the medium consisting of 15% FBS-containing DMEM medium supplemented with 10 µM SB239063 (Cayman Chemical, 19142) or 10 µM SB203580 (Cayman Chemical, 13067) or the solvent (Dimethyl Sulfoxide: DMSO, nacalai tesque, 09659-14) as a control, and P/S (1x). The medium was changed every 2 days. After the time described in the graph (12 h and 2–6 days, Figure 1B) in culture, the cultured cells were subjected to alkaline phosphatase (ALP) staining to count ALP-positive surviving PGCs. Images were captured with a microscope (Keyence, BZ-X710).

### Derivation of EGC and cEGLC line from developing PGCs

For the establishment of cell lines derived from PGCs, an isolated hindgut and genital ridge where PGC colonized were dissociated with 0.25% trypsin/1 mM EDTA for 10 min at 37°C and then plated on MMC-inactivated STO feeders in a single well of a 4-well plate in the medium described below (detailed media compositions are described in the Supplementary Table S1). DF15^2iLIF^ medium was used for EGC derivation, and DF15 plus p38 MAPK inhibitor (DK15^SB23^ or DK15^SB20^) medium was used for cEGLC derivation (see Supplementary Table S1). Medium was changed every 2 days. At 12–14 days after plating, the appeared Oct4-GFP-positive colony-forming cells were picked up, harvested, and then dissociated using TrypLE (Gibco, 12604-013), and transferred onto freshly prepared MMC-inactivated STO feeders for further cultivation. Established EGC and cEGLC lines were cultured on MMC-STO and passaged every 3–4 days at a split ratio of 1:20 in DK15 medium consisting of 15% Knockout Serum Replacement (KSR, Gibco, 10828028)-containing DMEM instead of FBS for better proliferation (supplemented with LIF for EGC instead of 2i compounds, with SB203580 for cEGLC-20 and with SB239063 for cEGLC-23 at the same concentrations as in the derivation of each cell line) (see Supplementary Table S1).

### Culture of mouse ESC and rsEpiSC lines

Established mouse ESC lines in ICR background were cultured on MMC-MEF in DF10^2iLIF^ (Okamura et al., 2021) (see Supplementary Table S1). Derivation and culture of rsEpiSC lines derived from E6.5 mouse epiblasts in ICR background were performed in chemically defined N2B27 medium supplemented with 20 ng/mL FGF2 (Peprotech, 100-18B) and 2.5 µM IWR-1 (Wnt inhibitor, Cayman Chemical, 13659) as described (Wu et al., 2015) (see Supplementary Table S1).

### Assays for signaling pathway with chemical inhibitors

For inhibition of signal transduction essential for the maintenance of pluripotency in conventional naïve and primed state, chemical compounds as potent and selective inhibitors with JAK inhibitor I (JAKi) (Janus kinase inhibitor, Millipore, 420099), SU5402 (Fibroblast growth factor receptor (FGFR) inhibitor; FUJIFILM, 197–16731), SB431542 (Activin pathway inhibitor, Cayman Chemical, 13031), LY294002 (PI3K inhibitor, Cayman Chemical, 70920) were administered at 1 µM (JAKi), 10 µM (SU5402), 10 µM (SB431542), 10 µM (LY294002), respectively, when the cells were passaged. After 4 days with compounds, the cultured cells were applied for immunofluorescence for OCT4 expression to be considered whether an undifferentiated state.

### Embryoid body formation for differentiation capacity

Dissociated 1 × 10^6^ cells (EGC and cEGLC-23) were plated on 35 mm petri dishes (Falcon, 351008) in “embryoid body formation medium” (EB formation medium, see Supplementary Table S1). After 4 days in culture, images were captured with a microscope (Olympus, CKX-53), and then embryoid bodies were processed for immunohistochemistry for GATA4 expression or for further differentiation. To demonstrate the ability to differentiate into three germ layers, formed embryoid bodies cultured in suspension for 4–5 days were transferred to a 0.2% gelatin-coated well in a single well of a 4-well plate for further attachment culture. After the embryoid bodies attached to the dish in “embryoid body attachment medium” (see Supplementary Table S1), the medium was changed to the media described below for further cultivation for 7 days. For ectoderm differentiation, “ectoderm differentiation medium” (see Supplementary Table S1) was used, and for mesoderm- and endoderm differentiation, “mesoderm and endoderm differentiation medium” (see Supplementary Table S1) was used, and prolonged cultured outgrowths of the attached embryoid body were then processed for immunohistochemistry to confirm the differentiation into three germ layers.

### Organ culture of isolated hindguts with p38 MAPK inhibitor

Hindguts encapsulating Oct4-GFP-expressing PGCs were isolated from Oct4-GFP mouse embryos at E8.75 and then plated on MMC-inactivated STO-feeder cells in “organ culture medium” (see Supplementary Table S1) supplemented with or without 10 µM SB239063 in a single well of a 4-well plate for hindgut outgrowth. After 8 days in culture, a hindgut outgrowth was scraped off with a pipette chip, dissociated with 0.25% trypsin/1 mM EDTA for 10 min at 37°C, and replated on MMC-inactivated STO-feeder cells in “organ culture medium” (see Supplementary Table S1) without SB239063. Oct4-GFP-positive surviving PGCs and colony-forming cells were observed under a microscope (Keyence, BZ-X710).

### Immunohistochemistry

For immunofluorescence studies, cells grown in a 4-well plate or cryosections of tissue were fixed with freshly prepared 4% paraformaldehyde in PBS for 15 min at room temperature and permeabilized/blocked with 1% Triton-X, 1% BSA, and 10% FBS in PBS for 1 h at room temperature. Cells and tissue sections were incubated with primary antibodies in 1% FBS, 1% BSA, and 0.1% Triton-X in PBS overnight at 4°C. The next day, the cells were washed three times in 0.1% Triton/PBS and incubated with fluorescence-labeled IgG (H + L) secondary antibodies (Abcam, goat anti-mouse_Alexa Fluor 488: ab150113, _Alexa Fluor 594: ab150116, goat anti-rabbit_Alexa Fluor 488: ab150077, _Alexa Fluor 594: ab150080, donkey anti-goat_Alexa Fluor 594: ab150132) at 1:500 dilution or incubated with HRP-conjugated goat anti-mouse IgG (H + L) (for brown staining) (Proteintech, SA00001-1) secondary antibody at 1:200 dilution in 1% FBS, 1% BSA, 0.1% Triton-X in PBS for 2 h at room temperature. Cells were washed three times, filled with 0.1% Triton/PBS, and nuclei were counterstained with DAPI (Sigma-Aldrich, D9542) for immunofluorescence. For brown staining, a peroxidase Staining DAB kit (nacalai tesque, 25985-50) was performed according to the manufacturer’s instructions. Primary antibodies used in this study include Anti-phosphorylated-p38 MAPK (p-p38 MAPK, diluted at 1:200; Cell Signaling Technology, 4511S), Anti-OCT4 (C-10) (diluted at 1:300, Santa Cruz, sc-5279), Anti-SOX-2 (E-4) (diluted at 1:300, Santa Cruz, sc-365823), Anti-OTX2 (diluted at 1:200, R&D systems, AF 1979), Anti-β-Tubulin III (diluted at 1:1000, Sigma-Aldrich, T2200), Anti-SMA/Actin/α-Smooth Muscle antibody (diluted at 1:600, Sigma-Aldrich, A5228), Anti-HNF-3β (diluted at 1:1000, Santa Cruz), Anti-GATA4 (G-4) (diluted at 1:250, Santa Cruz, sc-25310). For immunofluorescence analyses using the p-p38 MAPK antibody with migrating PGCs, analyses were performed as follows: Isolated hindguts were fixed in 4% paraformaldehyde in PBS overnight at 4°C, followed by incubation in 10% sucrose/PBS overnight at 4°C and then in 20% sucrose/PBS overnight at 4°C. Fixed tissues were embedded in OCT (Tissue-Tek) for cryosectioning at 10 μm, and staining was performed by immunofluorescence described above. Observation was made under a microscope (Keyence, BZ-X710).

### Alkaline phosphatase (ALP) staining

Cultured cells were fixed in 4% paraformaldehyde in PBS for 15 min at room temperature. The cells were washed three times in PBS and then stained with the ALP staining solution. The ALP staining solution was prepared as follows: 5 mg of naphthol AS-MX phosphate disodium salt (Sigma, N5000) was dissolved in 500 µL of N, N-Dimethylformamide, and then 10 mg of Fast Red TR Salt Hemi (Sigma, F8764) or Fast Blue RR salt (Wako, 069-03712) was dissolved in the above solution, and the solution was transferred to 9.5 mL of 0.1 M Tris-HCl (pH 9.5). In addition, the staining solution was filtered through a 0.2 µm filter to remove the formed precipitate and left on the fixed cells for more than 10 min at room temperature for better development. The stained cells were then washed three times in PBS and filled with PBS for observation under a microscope (Keyence, BZ-X710).

### Teratoma formation

2 × 10^6^ cells of EGCs or cEGLCs were resuspended in a 1:1 mixture of 100 µL of ice-cold PBS with 100 µL of Matrigel (Corning, 354234) and injected subcutaneously into male immunnodeficient nude mice (KSN/Slc) at 8–16 weeks of age to form teratomas. After 4 weeks, the mice were sacrificed by cervical dislocation, and teratomas were excised, fixed in 4% paraformaldehyde in PBS, embedded in paraffin, sectioned at 5 μm, and stained with hematoxylin-eosin for histological analysis.

### Generation of chimeric mice

To generate chimeric embryos, 10 cells from each EGC or cEGLC-23 line were injected into ICR background blastocysts, which were then transplanted into the uteri of pseudopregnant ICR females.

### RNA preparation and real-time PCR

Total RNA was extracted using RNeasy Mini Kit (QIAGEN, 74104) according to the manufacturer’s instructions. RNAs were reverse transcribed using ReverTra Ace qPCR RT Master Mix (TOYOBO, FSQ-201), and real-time PCR was performed using THUNDERBIRD SYBR qPCR Mix (TOYOBO, QPS-201) in MIC qPCR (bio molecular systems). The relative expression levels of each gene were normalized by Gapdh and calculated by the comparative CT method. Primer sequences are listed in Supplementary Table S2.

### RNA-sequencing and data analysis

All sequenced libraries were aligned to the mouse reference genome (UCSC mm10) using STAR (v2.7.0c) (Dobin et al., 2013). Hierarchical clustering analysis was performed using the R hclust function with Euclidean distance. Multi-dimensional scaling (MDS) analysis to show the variation among RNA-seq samples conducted by plotMDS function. Differential expression gene analysis was performed with edgeR (v3.32.1) (Robinson et al., 2009) package using gene count matrix generated by FeatureCounts (v.1.5.2) (Robinson et al., 2009). Sequencing libraries were prepared using the NEBNext Ultra II Directional RNA Library Prep Kit (NEB) and were sequenced on an Illumina Novaseq platform to generate 150 bp paired-end reads. The adaptors of the obtained raw sequence data were trimmed using Trim Galore (v0.6.3), and quality control using FastQC (v0.11.7). Processed reads were aligned to the mouse reference genome (UCSC mm10) using STAR (v2.7.0c) (Dobin et al., 2013). FeatureCounts v1.5.2 was used to generate the read-counting data for each gene. Differential gene expression analysis was performed using TCC (Sun et al., 2013; Tang et al., 2015). MDS plot analysis and Heatmap clustering were performed using the princomp, and heatmap packages from R.

### Gene enrichment analysis

KEGG pathway analysis was performed using clusterProfiler (Yu et al., 2012), and the connections between genes and biological concepts were displayed using the cnetplot function.

### Statistical analysis

Statistical analysis was performed using one-way and two-way ANOVA for multigroup comparison, when significant, group differences were evaluated using the Student’s t-test. *p* values <0.05 were considered as statistically significant.

## Results

### Inhibition of p38 MAPK in mouse migrating PGCs

To search for chemical compounds involved in PGC reprogramming, we particularly focused on molecular pathways that suppress pluripotency and teratoma formation in germ cells. Since reprogramming of PGCs could be involved in the onset of self-renewal as pluripotent stem cells, in this screening we took advantage chemical inhibitors that target signaling molecules or epigenetic modifiers that converts migrating PGCs into Oct4-GFP-positive colony-forming cells. We used the developing hindgut as a screening material: firstly because it encapsulates all developing PGCs, which is important to avoid bias due to heterogeneity of PGCs; and secondly because the proliferation of surrounding somatic cells after plating into culture dishes is far less than other tissues encapsulating the developing PGCs such as emerging gonads, thereby resulting in easy detection of colony formation of reprogrammed PGCs. We screened approximately 50 different compounds at the various concentrations to monitor induction of colony-forming cells derived from cultured dissociated hindguts of Oct4-GFP embryos at E8.75. This led to identification of SB239063, a p38 MAPK inhibitor, as a candidate chemical compound (Figure 1A). After day 2 in culture, PGCs positive for ALP activity increased and the number of these cells peaked out. Of note, administration of SB239063 in culture significantly supported the survival or proliferation of PGCs (Figure 1B). Furthermore, ALP- and Oct4-GFP-positive cells formed clusters after 4 days in culture in the presence of SB239063, while Oct4-GFP expressing cells were dispersed in control media (Figures 1B,C). After around 12 days in culture, some colony-forming cells expressing Oct4-GFP appeared only when treated with SB239063, and the colonies subsequently expanded in size (Figure 1D). Assuming that the colony-forming cells were derived from PGCs, activated p38 MAPK protein was likely expressed in migrating PGCs in the developing hindgut and may therefore have some function, particularly in inhibiting reprogramming of PGCs to pluripotent stem cells. Consistent with this notion, phosphorylated p38 MAPK protein was clearly and exclusively detected in a sub-population of migrating Oct4-GFP-expressing PGCs at E8.75, but not in surrounding somatic cells (Figure 1E).

In addition to the E8.75 embryo hindguts, we attempted to establish cEGLCs using emerging gonads encapsulating PGCs. However, no Oct4-GFP-positive colony-forming cells emerged from the dissociated emerging gonads at E11.5 and E12.5 in both males and females, cultured under the same conditions with hindguts (Supplementary Table S3).

### Pluripotency of cEGLCs in naïve state

**FIGURE 2:**
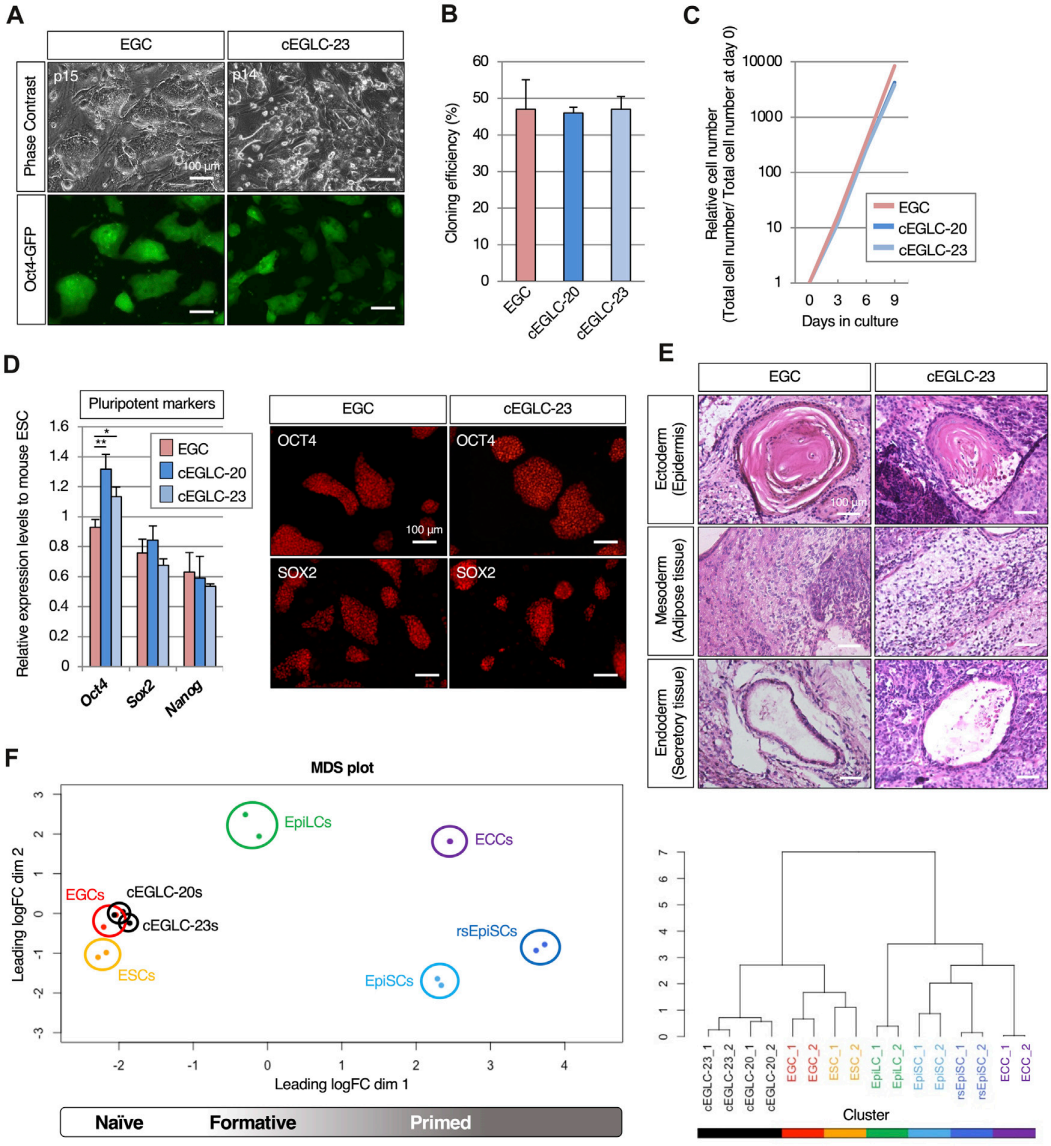
Pluripotency of cEGLC in naïve state. **(A)** Images of Oct4-GFP-positive EGCs (Embryonic Germ Cells) and cEGLCs-23 (chemically induced EG-like cells with SB239063) at the indicated passage number in images. Scale bar, 100 μm. **(B)** Cloning efficiency in EGC, cEGLCs-20 (chemically induced EG-like cells with SB203580) and cEGLCs-23. Error bars indicate s.d. (*n* = 3, independent experiments). **(C)** Graphical representation of relative cell number in proliferation of cultured EGC, cEGLC-20 and -23. The ANOVA test reveals that there is no significant difference among the three groups (*p* = 0.6577). **(D)** Quantitative PCR analysis of expression of pluripotent markers in EGCs, cEGLC-s20 and -23. Error bars indicate s.d. (*n* = 3, biological replicates). *t*-test, **p < 0.01. *p < 0.05. Images of OCT4 (upper) and SOX2 (bottom) protein expression (pluripotent markers) in colonies of EGCs and cEGLCs-23. Scale bar, 100 μm. **(E)** Representative images of hematoxylin and eosin-stained sections of the subcutaneously formed teratoma. Scale bar, 100 μm. **(F)** Bioinformatic analysis of RNA-sequencing data with mouse pluripotent stem cells including cEGLCs. MDS (Multidimensional scaling) plot and hierarchical clustering dendrogram under optimal rank (c) based on differentially expressed genes (DEGs) among 8 cell types. Replicates of the same conditions are indicated by same color, cEGLCs-20 and −23 (black), ESCs (orange), EGCs (red), EpiLCs (green line, published data set (Shirane et al., 2016)), ECCs (P19) (purple, published data set (Dai et al., 2022)), EpiSCs (light blue) and rsEpiSCs (blue) published data set (Wu J. et al., 2015).

SB203580, another p38 MAPK chemical inhibitor, also induced Oct4-GFP-expressing colony-forming cells in the same culture system using isolated hindgut (Supplementary Figure S1A). Picked-up colonies of Oct4-GFP-expressing cells could be maintained long-term by passaging with trypsinization (Figure 2A and Supplementary Figure S1B). This indicated that derivation of pluripotent stem cell lines could be performed under novel conditions using p38 MAPK inhibitors. Cell lines derived with SB239063 or SB203580 were named chemically induced Embryonic Germ-like Cell with SB239063 (cEGLC-23 for abbreviation) or cEGLC-20, respectively.

Surprisingly, cEGLCs-23 and -20 could be maintained long-term as Oct4-GFP-expressing cells without LIF supplementation (Figure 2A and Supplementary Figure S1B), whereas EGCs derived from migrating PGCs continuously required LIF (Figure 2A). EGCs displayed a dome-shaped colony morphology that is a representative characteristic of naïve pluripotency, whereas cEGLCs exhibited a more spread and flattened colony morphology indistinguishable from STO-feeders until 2 days after passage, suggesting that cEGLCs may not possess representative characteristics of naïve pluripotency (Figure 2A). However, even without Y-27632, a ROCK inhibitor, the cloning efficiency in cEGLCs was equivalent to that in EGCs (Figure 2B). In addition, there were no significant differences between EGC and cEGLC lines in proliferation rate (Figure 2C) and expression levels of pluripotent markers in terms of transcription and protein (Figure 2D and Supplementary Figure S1C). The ability of the cEGLC lines to differentiate into three germ layers was confirmed by induction of embryoid body differentiation (Supplementary Figure S2) and teratoma formation (Figure 2E and Supplementary Figure S1D). The significance of our analysis of teratoma histology is that it demonstrates the multipotent nature of cEGLCs and thus the reprogramming of migratory PGCs, which are monopotent. In fact, the teratoma tissue was not a simple histological picture, such as seminomas, yolk sac tumors or even choriocarcinomas, which are observed during tumorigenesis of germ cell origin (Barrios et al., 2013), but a complex structure containing mature and diverse tissue, which confirms the multipotent and reprogrammed status of cEGLCs (Figure 2E).

To compare gene expression profiles of cEGLCs with naïve or primed mouse pluripotent stem cells, we performed RNA-sequencing. Unsupervised MDS plot analysis revealed that cEGLCs were closely clustered with ESCs and EGCs (naïve) but not with EpiLCs (formative), EpiSCs, or rsEpiSCs (primed) (Figure 2F_left panel). Unlike other stem cells, P19 embryonal carcinoma cells (ECCs) derived from teratocarcinoma can proliferate indefinitely and maintain an undifferentiated state without external signals. ECCs were also clearly distinguished from cEGLCs. Hierarchical clustering analysis, however, showed that cEGLCs are moderately different from ESCs and EGCs (Figure 2F_right panel). These results indicate that cEGLC lines display the characteristics of naïve pluripotency like those of ESCs and EGCs despite their distinct colony morphology and moderate differences in gene expression.

### Presence of both epiblast-like and naïve-like features in cEGLCs

**FIGURE 3:**
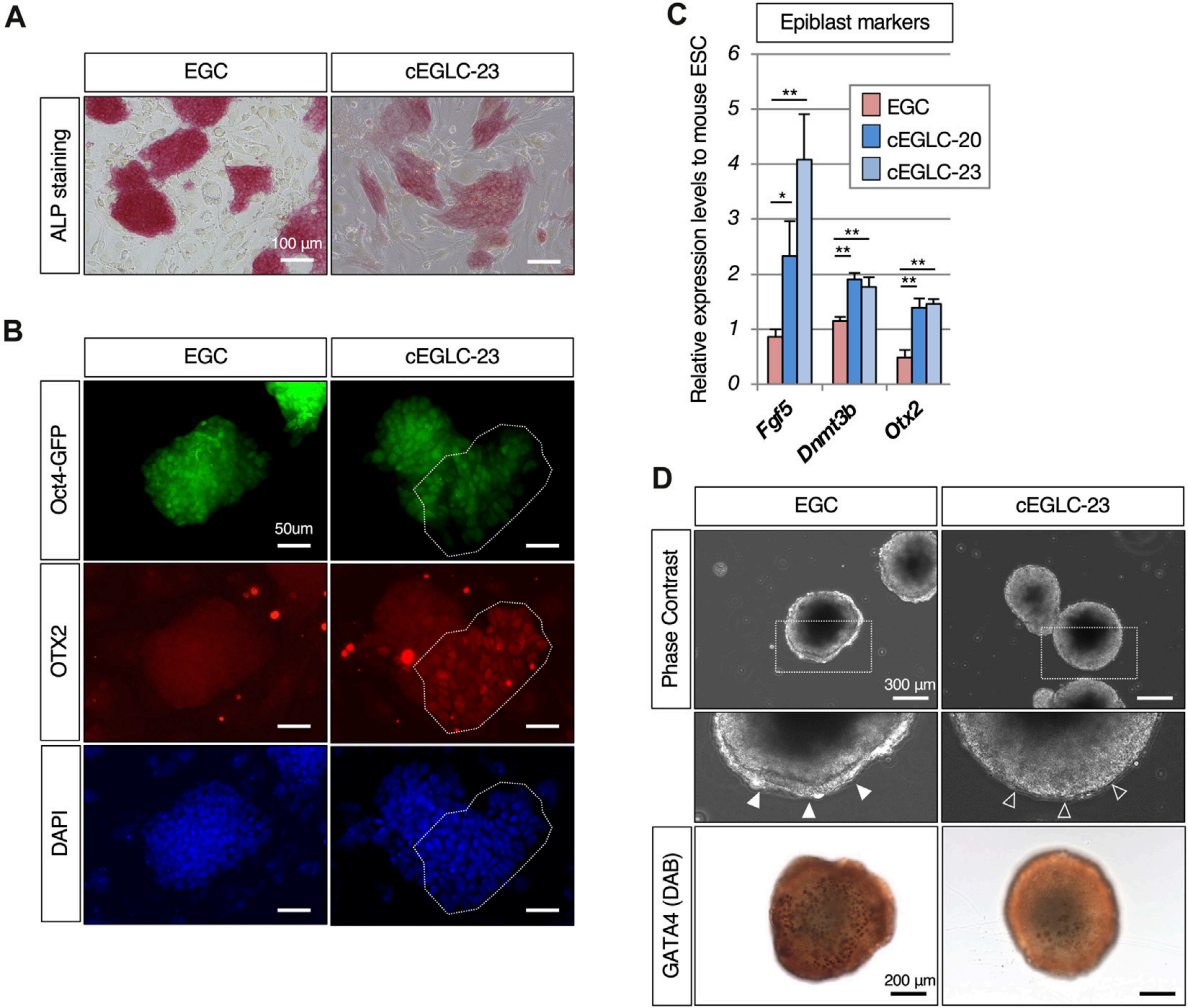
Epiblast-like characteristics in cEGLCs despite naïve state. **(A)** Differential intensity of alkaline phosphatase (ALP) activity between EGCs and cEGLCs-23. Scale bar, 100 μm. **(B)** Immunofluorescence images of OTX2 protein expression (a definitive marker of the epiblast) in colonies of EGCs and cEGLCs-23. Nuclei were counterstained with DAPI. The encircling dotted lines indicate a portion of cells in the colony of cEGLCs-23 that are clearly expressed for OTX2 protein. Scale bar, 50 μm. **(C)** Quantitative PCR analysis of expression of epiblast markers in EGCs, cEGLCs-20 and −23. Error bars indicate s.d. (*n* = 3, biological replicates). *t*-test, **p < 0.01. *p < 0.05. **(D)** Analysis of definitive endoderm formation in embryoid bodies. Representative images showing the formation of embryoid bodies derived from EGCs and cEGLCs-23 on day 4 in culture. Middle images correspond to square dotted lines in top images at higher magnification. White filled and empty arrowheads indicate a thick and no apparent layer of primitive endoderm respectively. Immunohistochemistry images for GATA4 protein expression detected by DAB staining in embryoid bodies derived from EGCs and cEGLCs-23. Scale bar in top and bottom images, 300 and 200 µm respectively.

To determine specific features of cEGLCs, we examined expression of marker genes and performed functional analysis to characterize the difference between naïve and primed states, given that the colony morphology of cEGLCs was not dome-shaped which is a representative feature of the naïve state (Figure 2A and Supplementary Figure S1B). We found that both cEGLC-23 and -20 lines exhibited characteristics differences from naïve pluripotency, namely, lower intensity of ALP activity (Figure 3A and Supplementary Figure S1E), partial but clearly detected OTX2 protein, a definitive marker of epiblast (Figure 3B and Supplementary Figure S1F), and higher expression levels of epiblast markers (Figure 3C). To assess the differentiation potential of cEGLCs into the primitive endoderm, a representative feature of naïve pluripotency, we performed embryoid body analysis (Figure 3D). cEGLCs-23 cells showed a limited ability to form an outer layer of GATA4-positive primitive endoderm in embryoid bodies, which was in sharp contrast to naïve EGCs. Taken together, these results indicate that cEGLCs closely resemble other naive pluripotent stem cells (ESCs and EGCs) in terms of their broad gene expression profiles (Figure 1F), but they also possess partial features of the epiblast that are not normally seen in naïve pluripotency.

### Alternative signaling requirement for pluripotency in cEGLCs

**FIGURE 4:**
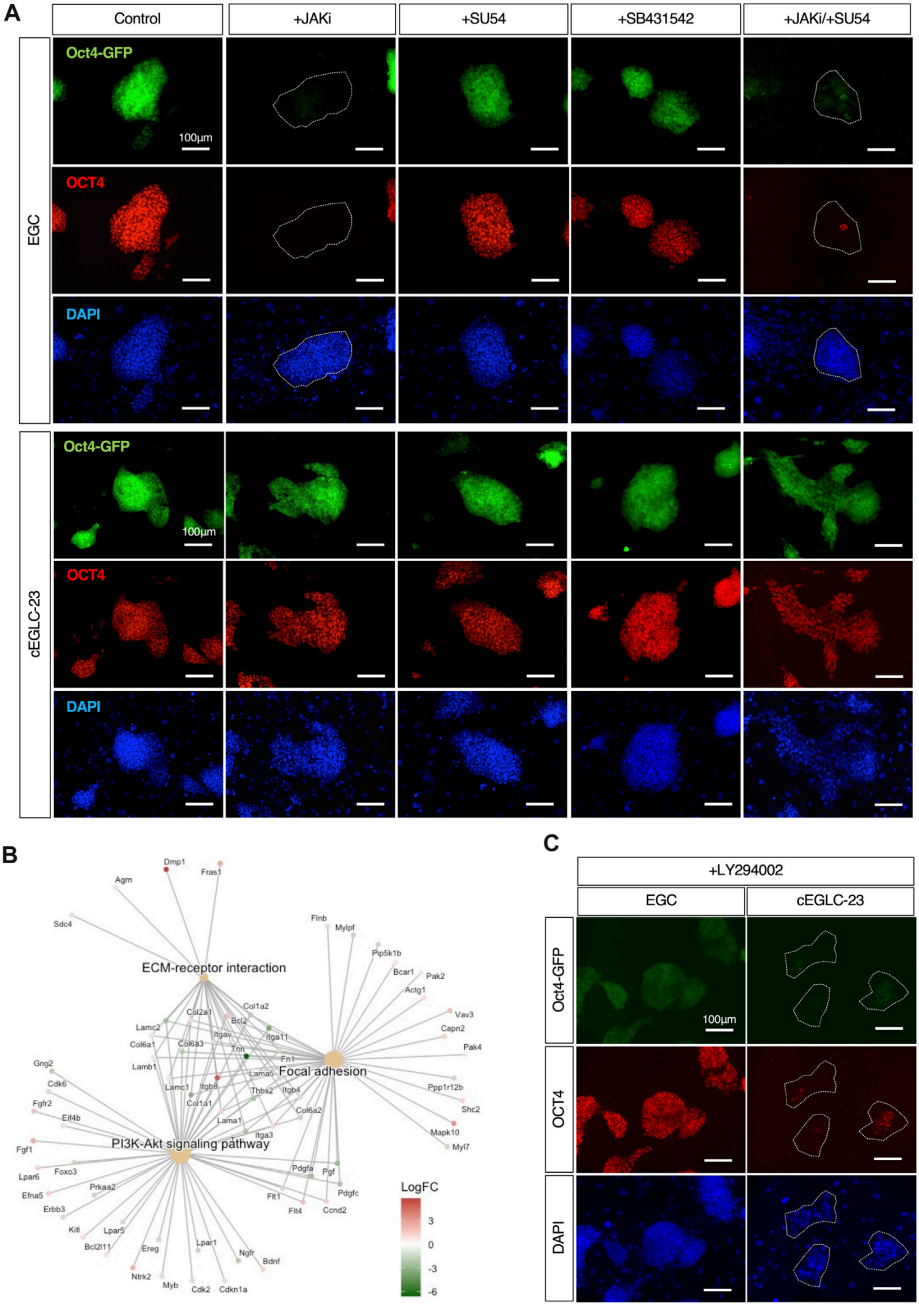
Differential signaling requirements for maintenance of pluripotency between EGCs and cEGLCs. **(A)** Representative images of Oct4-GFP and OCT4 protein expression (markers for the undifferentiated state) in colonies of EGCs, cEGLCs-23 on day 4 with administration of drugs, JAKi (A Janus kinase inhibitor), SU54 (FGFR inhibitor), SB431542 (TGF-β/Activin inhibitor), a combination of JAKi/SU54. Nuclei were counterstained with DAPI. The white circled dotted line indicates colonies of EGCs that lose OCT4 expression. Scale bar, 100 μm. **(B)** Gene Set Enrichment Analysis (GSEA) of DEGs between ESC-EGC and cEGLCs groups. Cnetplot shows enriched KEGG (Kyoto Encyclopedia of Genes and Genomes) pathways. **(C)** Representative images of Oct4-GFP and OCT4 protein expression in colonies of cEGLCs-23 on day 4 with LY294002, a PI3K kinase inhibitor. Nuclei were counterstained with DAPI. The white circled dotted line indicates colonies of cEGLCs-23. Scale bar, 100 μm.

Since cEGLCs partially exhibited epiblast-like features, albeit in a naïve state, we investigated the intracellular signaling pathways involved in maintaining pluripotency. Withdrawal of LIF and inhibition of JAK/STAT3 signaling rapidly induce differentiation in mouse ESCs, reconfirming the requirement of LIF-JAK signaling dependence in the naïve pluripotency (Boeuf et al., 1997; Niwa et al., 1998). Similarly, inhibition of FGF2 and Activin A pathways in primed pluripotent EpiSCs induced cell death and neuroectodermal differentiation (Greber et al., 2010; Wu J. et al., 2015). To determine the signaling pathways for maintaining pluripotency in cEGLCs, we exposed these cells to specific inhibitors of JAK (JAKi), FGF2 (SU5402), or Activin A (SB431542). Four days after inhibition of JAK, but not FGF2 or Activin A, most EGC colonies exhibited loss of OCT4 protein expression and decrease of Oct4-GFP signals (Figure 4A). In contrast, OCT4 levels remained unchanged in cEGLCs (Figure 4A). Considering the possibility of switching of the dependency on signaling pathway during culture, we also checked the effect of combined inhibition of JAK and FGF pathways (Figure 4A). Even after treatment with dual inhibitors, however, cEGLCs showed stable expression of OCT4, indicating that cEGLCs maintain pluripotency independent of conventional signaling pathways such as JAK or FGF2.

### Requirement of the PI3K-Akt signaling for cEGLC pluripotency

To determine the signaling pathways required for pluripotency in cEGLCs, we performed Gene Set Enrichment Analysis (GSEA) of DEGs (Differential Expressed Genes) between the ESCs-EGCs group and cEGLCs group (Figure 4B). Visualization of results with “Cnetplot,” based on the correlation between DEGs and GO terms, indicated that PI3K-Akt signaling pathways, focal adhesion, and ECM-receptor interaction could be highly activated in cEGLCs.

The PI3K (phosphoinositide 3-kinase) signaling pathway, which is activated by growth factors and cytokines including LIF, is important for proliferation, survival, and maintenance of pluripotency in mouse ESCs (Watanabe et al., 2006). To test whether the PI3K pathway is essential for pluripotency in cEGLCs, or whether there is a difference in sensitivity to PI3K pathway inhibition between EGCs and cEGLCs, exposure to a specific inhibitor of PI3K-Akt signaling (LY294002) was performed. Upon treatment with LY294002 for 4 days, most cEGLC-23 colonies lost OCT4 protein and Oct4-GFP expression, whereas EGCs were barely affected (Figure 4C). We speculate that in cEGLCs, activation of the PI3K-Akt pathway as a mechanism for pluripotency may utilize unknown exogenous factors rather than well-known ones such as LIF and FGF2/Activin A (Figure 4A), or may utilize self-activated endogenous mechanisms independent of p38 MAPK inhibition (Supplementary Figure S3).

The increased expression of genes related to focal adhesion and ECM (extracellular matrix)-receptor interaction may correlates with changes in the cell’s adherence to the surrounding matrix (Figure 4B). This can influence the physical morphology of colonies (Figure 2A and Supplementary Figure S1B). Increased focal adhesion can lead to a more spread and flattened colony morphology due to stronger interactions with the ECM (see discussion).

### 2iLIF supplementation elicits competence for chimera formation in cEGLCs

**FIGURE 5:**
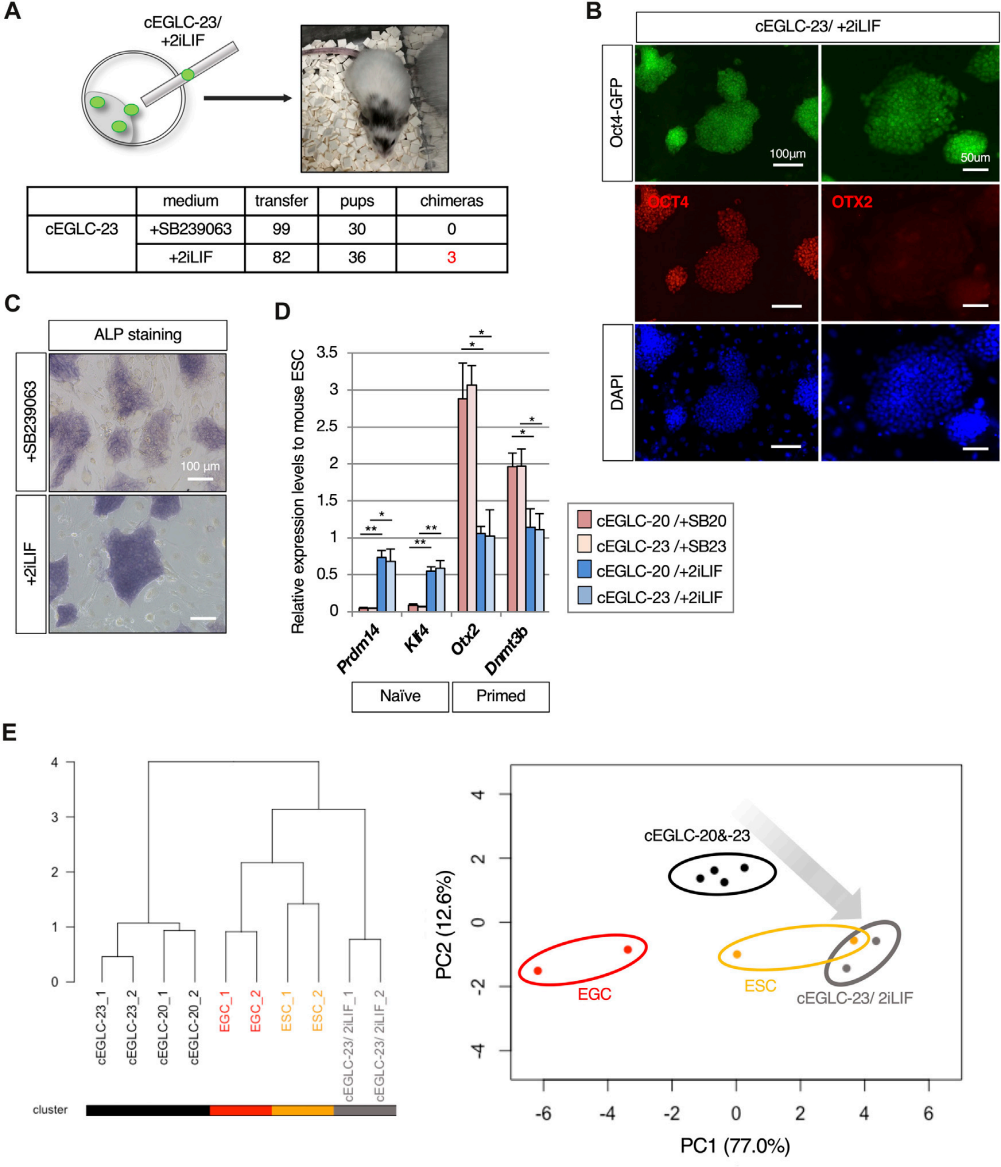
Supplementation of 2iLIF elicits the competence of chimera formation in cEGLCs. **(A)** Chimera formation analysis by blastocyst injection with cEGLCs-23 in different culture conditions. Schematic of the blastocyst injection with cEGLC-23 adapted in 2iLIF supplementation, and image of mouse pup showing the partially coat color-altered chimera in the head and tail. Table for chimera formation efficiencies in different media conditions with the actual number performed. **(B)** Images of Oct4-GFP expression and immunofluorescence of OCT4 and OTX2 protein expression in cEGLCs-23 adapted with 2iLIF supplementation. Nuclei were counterstained with DAPI. Scale bar in left and right images, 100 and 50 µm respectively. **(C)** The effect of 2iLIF supplementation on alkaline phosphatase (ALP) activity compared with that of SB239063. Scale bar, 100 μm. **(D)** Quantitative PCR analysis of naïve and epiblast markers expression in cEGLCs treated with SB239063 or 2iLIF. Error bars indicate s.d. (*n* = 3, biological replicates). *t*-test, **p < 0.01. *p < 0.05. **(E)** Bioinformatic analysis of RNA-sequencing data. (left panel) The dendrogram with all detected genes shows the hierarchical relationship between RNA-sequencing samples across cell lines and culture conditions. (right panel) PCA plot based on differentially expressed genes (DEGs) among cell types and different cultured conditions. cEGLCs-20 and -23 maintained treated with each of p38 MAPK inhibitors (circled with black line), cEGLCs-20 and −23 adapted with 2iLIF supplementation (circled with gray line), ESCs (circled with orange line) and EGCs (circled with red line).

A typical method for classifying naïve and primed pluripotency is chimera formation by blastocyst injection, since injection of primed pluripotent stem cells such as mouse EpiSCs into the blastocyst does not result in the formation of chimeras (Mascetti and Pedersen, 2016). As described above, cEGLCs exhibit naïve pluripotency (Figure 2), along with partial epiblast-like features, including expression of protein (Figure 3B) and certain genes (Figure 3C) and lacking the ability to differentiate into primitive endoderm during embryoid body formation (Figure 3D). Intriguingly, chimera formation assay showed that cEGLCs-23 cultured with 2iLIF formed chimeras, but those cultured with SB239063 (DK15^SB23^) did not (Figure 5A), indicating that cEGLCs-23 may have a latent ability to form chimeras and that SB239063 likely blocks this ability.

To confirm the molecular features underlying the ability to form chimeras, we analyzed expression patterns of pluripotent markers and transcriptional profiles of cEGLCs cultured with 2iLIF (cEGLC-23/2iLIF). As shown in Figures 5B–D, cEGLCs cultured with 2iLIF exhibited a loss of epiblast-like features such as OTX2 protein expression (Figure 5B), intense ALP activity (Figure 5C), and transcriptional adaptation into naïve pluripotency representing increased naïve and decreased primed marker genes (Figure 5D). Consistent with these results, analysis of RNA-seq data also suggested that the gene expression profiles of cEGLCs-23 cultured with 2iLIF (cEGLC-23/2iLIF) are closer to naïve pluripotent states as observed in EGCs/ESCs (Figure 5E).

### *Ex vivo* analysis of p38 MAPK inhibition to measure teratoma formation capacity

**FIGURE 6:**
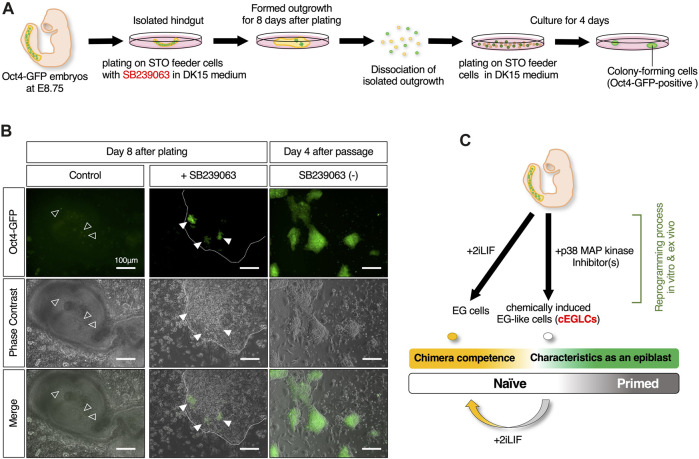
*Ex vivo* analysis of reprogramming competence in migrating PGCs by inhibition of p38 MAPK. **(A)** Schematic of the experimental workflow for induction of *ex vivo* reprogramming of migrating PGCs in an isolated hindgut encapsulating Oct4-GFP expressing PGCs is isolated from Oct4-GFP mouse embryo at E8.75 with SB239063. **(B)** Image of the outgrowth of plated hindgut encapsulating the dispersed Oct4-GFP-expressing PGCs in control (the empty arrowheads in left images) and those clustered in SB239063-treated (the filled arrowheads in middle images). Images of Oct4-GFP-positive colony-forming cells appeared after dissociated replating even after withdrawal of SB239063 (right images). The edge of plated hindgut outgrowth is depicted with dotted outline. Scale bar, 100 μm. **(C)** Schematic summary of *in vitro* and *ex vivo* reprogramming of mouse migratory PGCs with p38 MAPK inhibitor(s) for cEGLC derivation.

To determine whether p38 MAPK is the molecular signal that suppresses pluripotency and teratoma formation in germ cells, we examined isolated hindguts, in which migrating PGCs are closely associated with the surrounding somatic cells, as an *ex vivo* organ culture model for teratoma formation. Hindguts encapsulating Oct4-GFP-expressing PGCs were isolated from Oct4-GFP mouse embryos at E8.75, and plated on MMC-inactivated STO-feeder cells without dissociation in the presence or absence of SB239063 (Figure 6A). In the control media, some of the surviving Oct4-GFP-expressing PGCs were dispersed but not clustered in the outgrowth of the plated hindgut, (Figure 6B). In the media supplemented with SB239063, several clusters consisting of cells positive for Oct4-GFP expression were formed in the outgrowth of hindgut after 6–8 days. To determine the acquirement of indefinite proliferative potential, after 8 days in culture, hindgut outgrowths were scraped off by pipette tips, followed by cell dissociation by trypsinization and replating in organ culture media without SB239063. Even when SB239063 was removed, Oct4-GFP-positive colony-forming cells appeared and grew continuously. These results, therefore, suggest that PGCs, even within the surrounding somatic cells, are reprogrammed by suppression of p38 MAPK activity in organ culture, and acquire indefinite proliferative potential, which may be the putative origin of teratoma development. Our culture system, therefore, could be considered as an *ex vivo* organ culture model to probe teratoma formation.

## Discussion

The present study provides definitive evidence that in mice the broad developmental potential of migratory PGCs is restricted by p38 MAPK. Pluripotency exerted by suppression of p38 MAPK, exhibits characteristics reminiscent of naïve pluripotency but with epiblast features and independence from conventional signaling pathways such as LIF or FGF2/Activin-A.

p38 MAPK is activated in response to various stress signals, including DNA damage, oxidative stress, and cytokines. Once activated, p38 MAPK can induce cell cycle arrest (Faust et al., 2012) or apoptosis (Huang et al., 2023), effectively eliminating potentially cancerous cells. Furthermore, p38 MAPK signaling initiates G1 mitotic arrest not only in cancer cells but also in a variety of terminally differentiated cell types (Perdiguero et al., 2007; Molnár et al., 1997; Xiu et al., 2003). In this study, PGCs cultured with SB239063, a p38 MAPK inhibitor, showed enhanced cell proliferation and suppression of cells death (Figures 1B,C) (Pesce and De Felici, 1994). Bcl-x, a member of the Bcl-2 family, and Bax proteins regulate the survival and apoptosis of mouse PGCs during embryogenesis (Rucker et al., 2000), and a subpopulation of migrating PGCs are positive for phosphorylated p38 MAPK protein (Figure 1E). As p38 MAPK-induced BAX/Bcl-2/caspase-3 apoptosis signaling is involved in glioma cell proliferation (Huang et al., 2023), p38 MAPK may play a pivotal role in controlling PGCs output by eliminating damaged or unscheduled pluripotency expression through mediation with Bcl-x and Bax proteins. In considering another potential role of p38 MAPK in reprogramming, it is noteworthy that the PI3K-Akt signaling pathway is highly activated in cEGLCs and that they are more strongly dependent on its signaling than conventional EGCs in maintaining undifferentiated state (Figures 4B,C). Activation of the MKK6-p38 MAPK pathway has been shown to negatively regulate downstream PI3K-Akt signaling through the suppression of IRS-1 and IRS-2 expression in 3T3-L1 adipocytes (Fujishiro et al., 2003). Given that Akt activation enhances PGC reprogramming into EGCs (Nagamatsu et al., 2012; Kimura et al., 2003; Matsui et al., 2014), inhibition of p38 MAPK within cultured PGCs may cause the activity of PI3K-Akt signaling, which in turn may trigger PGC reprogramming into cEGLCs.

The p38 MAPK signaling pathway plays a crucial role in the development of germ cells in embryonic gonads, and is particularly involved in the sexual differentiation of XY PGCs in mice (Ewen et al., 2010; Wu Q. et al., 2015). Research indicates that p38 MAPK signaling is essential for preventing the meiotic entry of XY PGCs, promoting their differentiation along the male pathway. Consistent with its functions, the expression of phosphorylated p38 MAPK, which is also found in surrounding somatic cells at E11.5, was exclusively observed in most of the germ cells in fetal testis at E13.5 (Wu Q. et al., 2015). In this study, the p38 MAPK in migratory PGCs play a crucial role as a gatekeeper of reprogramming, and absence of its function results in derivation of pluripotent stem cells *in vitro* (Figures 1, 2) and teratoma-like colony-forming cells *ex vivo* (Figure 6). Taken together, the functions of p38 MAPK in PGCs are likely to vary depending on the environment in which PGCs localize and their developmental stage, even within a narrow window from migration to gonadal stages.

Embryonal carcinoma cells (ECCs) are considered as the stem cells of teratocarcinoma, a type of germ cell tumor. They share many similarities with embryonic stem cells (ESCs) in terms of their pluripotency and the ability to differentiate into various cell types (Kelly and Gatie, 2017). One of the remarkable features of ECCs is their capacity to maintain pluripotency, because unlike ESCs, ECCs can maintain pluripotency in the absence of LIF or feeder layers (Kawazoe et al., 2009; Martin, 1981). Unlike mouse ESCs and EpiSCs, which typically require the presence of specific growth factors and cytokines in the culture medium to maintain their undifferentiated state (such as LIF for mouse ESCs and FGF2/Activin-A for mouse EpiSCs), cEGLCs exhibit a certain level of independence from these exogenous supplements (Figure 4A). This independence suggests that cEGLCs may possess novel intrinsic, or autocrine, or extrinsic signaling mechanisms that can maintain pluripotency similar to ECCs (Kawazoe et al., 2009), and may involve activation of downstream of the PI3K-Akt signaling pathway (Figures 4B,C). It has been clearly demonstrated that Akt signaling sufficiently maintains pluripotency in mouse and primate ESCs (naïve and primed), supporting the notion that the PI3K-Akt signaling axis regulates “stemness” in a broad spectrum of stem cell systems, regardless of differential requirements for external growth factors or cytokines (Watanabe et al., 2006).

Enrichment analysis of RNA-sequencing data from the ESC-EGC and cEGLC groups showed that PI3K-Akt, focal adhesion, and ECM-receptor interaction are the pathways that are more enriched in the cEGLC group (Figure 4B). Furthermore, chemical inhibition by PI3K inhibitor resulted in more sensitive effects on Oct4-GFP and OCT4 protein expression in cEGLCs (Figure 4C), suggesting that cEGLCs are more dependent on the PI3K-Akt pathway for maintaining undifferentiated status than the ESC-EGC group. Moreover, enrichment of focal adhesion and ECM-receptor interaction in cEGLCs may successfully explain a more spread and flattened colony morphology characteristic of cEGLCs (Figure 2A and Supplementary Figure S1B). Pluripotent stem cells such as human iPSCs (Närvä et al., 2017) and mouse ESCs (Taleahmad et al., 2017) with activated focal adhesion and ECM-receptor interaction genes exhibit different physical interactions with the surrounding ECM, leading to a more spread and flattened colony morphology due to stronger interactions with the ECM.

One of the remarkable features of cEGLCs is that they are naïve pluripotent states with epiblast features, which are representatively characterized by the expression of the OTX2 protein in a subset of the cell population within the cell colony (Figure 3B). One aspect of this feature is that while cEGLCs do not show the potential for chimaera formation under standard culture conditions without growth factors or compounds other than p38 MAPK inhibitors, they can exhibit potential chimera formation ability when induced into an earlier stage of development, namely, the ground naïve state, under 2iLIF conditions (Figure 5). These results suggest that alterations in epigenetic modifications or metabolic signatures in cEGLCs compared to EGCs may play a role in representative features of cEGLCs described above that differ from conventional naïve pluripotent cells such as ESCs and EGCs (Andrews et al., 2005). Considering the positioning of cEGLCs within the spectrum of pluripotency, cEGLCs exhibit a novel pluripotent entity of being naïve state with masked developmental capabilities that could be exerted by 2iLIF treatment, and regulatory mechanisms distinct from conventional pluripotent states (Figure 6C).

To further characterize the unique pluripotency of cEGLCs, it's pivotal to note that previously derived pluripotent stem cells from migratory PGCs exhibited naïve characteristics (Matsui et al., 1992; Resnick et al., 1992; Leitch et al., 2010), possibly reflecting PGCs’ intrinsic pluripotent state. However, the universal addition of LIF, which is essential for maintaining naïve pluripotency, in establishing EGCs hints that cEGLCs’ distinct pluripotency with epiblast features might arise from their establishment without LIF. This scenario presents two potential origins of cEGLCs’ pluripotency: an artificial state induced by p38 MAPK inhibition or a default pluripotent state inherent to migratory PGCs, warranting further investigation.

Somatic cells can be reprogrammed into iPSCs by the introduction of the transcription factors Oct4, Sox2, Klf4, and c-Myc (OSKM) (Takahashi and Yamanaka, 2006; Takahashi et al., 2007), and PGCs can be reprogrammed into pluripotent stem cells known as EGCs with FGF2/LIF/SCF supplementation (Matsui et al., 1992; Resnick et al., 1992; Shamblott et al., 1998). They share many similarities in terms of regulatory processes and signaling involved in reprogramming (Sekita et al., 2016). The tumor suppressor Trp53 is a gatekeeper that checks the balance between proliferation and apoptosis, and its inhibition plays a crucial role in the reprogramming of cultured PGCs as downstream targets of the PI3K-Akt (Kimura et al., 2008). On the other hand, deletion or knockdown of *Trp53* also greatly enhances iPSC induction (Krizhanovsky and Lowe, 2009). Furthermore, treatment with 2i (PD0325901 and CHIR99021) and Akt activation commonly enhances the efficiency of OSKM-induced iPSC derivation (Shi et al., 2008; Silva et al., 2008; Tang et al., 2014; Yu et al., 2014) and PGC reprogramming into EGCs (Nagamatsu et al., 2012; Kimura et al., 2003; Matsui et al., 2014). It has been shown in this study that p38 MAPK inhibition by chemical compounds give rise to reprogramming of migratory PGCs for cEGLC derivation (Figure 1), having in common with application of specific p38 MAPK inhibitors increases the number of mouse iPSC colonies (Li and Rana, 2012).

The *ex vivo* experiments conducted in this study further substantiate the pivotal role of p38 MAPK in the regulation of PGC fate. By employing an organ culture system with isolated hindguts encapsulating migrating PGCs, we demonstrated that inhibition of p38 MAPK not only promotes survival and proliferation of PGCs but also potentially reprograms them, endowing these cells with indefinite proliferative capabilities (Figure 6). More importantly, clusters of Oct4-GFP expression-positive cells formed by culturing E8.75 hindgut in SB239063 appear after 6–8 days of culture (Figure 6B), whereas teratoma formation occurs at a high frequency in the developing testis of 129/Sv inbred mouse strains (Stevens, 1980) and *Pten*-deficient mice (Kimura et al., 2003), where their teratoma foci formation is observed at the E15.5. This result implies that our proposed *ex vivo* experimental system can faithfully mimic teratoma formation *in vivo*, and therefore, these results illustrate a direct link between the molecular inhibition of p38 MAPK and the acquisition of a reprogrammed state capable of teratoma formation.

This comprehensive study elucidates the pivotal roles of p38 MAPK signaling in the fate and plasticity of PGCs, underscoring its gatekeeping function in the prevention of unscheduled cell fate transitions and maintenance of cellular integrity. The discovery that p38 MAPK activity confine the broad developmental potential of PGCs, safeguarding against aberrant gametogenesis and teratoma formation, provides significant insight into the molecular underpinnings of germ cell development and pluripotency regulation. This study not only advances our understanding of latent pluripotency in germ cells but also opens new avenues for therapeutic interventions in germ cell tumor such as teratoma.

## Data Availability

The datasets presented in this study are available in online repositories. The data have been deposited with links to BioProject accession number PRJDB17795 in the DDBJ BioProject database (https://www.ncbi.nlm.nih.gov/bioproject/?term=PRJDB17795).
